# The Effectiveness of Continuous Glucose Monitoring Devices in Managing Uncontrolled Diabetes Mellitus: A Retrospective Study

**DOI:** 10.7759/cureus.42545

**Published:** 2023-07-27

**Authors:** Andre E Manov, Sukhjinder Chauhan, Gundip Dhillon, Athena Dhaliwal, Sabrina Antonio, Ashrita Donepudi, Yema N Jalal, Jonathan Nazha, Melissa Banal, Joseph House

**Affiliations:** 1 Internal Medicine, Sunrise Health Graduate Medical Education (GME) Consortium, Las Vegas, USA; 2 Internal Medicine, MountainView Hospital, Las Vegas, USA; 3 Anesthesiology, MountainView Hospital, Las Vegas, USA; 4 Radiology, MountainView Hospital, Las Vegas, USA

**Keywords:** glycemic control, uncontrolled diabetes mellitus, continuous glucose monitoring, board certified endocrinologist, internal medicine residents, self-monitoring blood glucose (smbg), hba1c, continuous glucose monitoring (cgm), diabetes mellitus type 2

## Abstract

This retrospective study aimed to assess the effectiveness of continuous glucose monitoring (CGM) devices in managing uncontrolled diabetes mellitus (DM). The study cohort comprised 25 patients with uncontrolled diabetes who received treatment at an internal medicine resident clinic. The objective was to evaluate the impact of transitioning from self-monitoring of blood glucose (SMBG) to CGM devices on glycemic control, as measured by changes in hemoglobin A1c (HbA1c) levels, average blood glucose levels, hypoglycemic events, time spent within the target blood sugar range, and glucose variability. The findings indicated significant improvements in glycemic control with the adoption of CGM devices, highlighting their potential benefits for optimizing diabetes management.

The study is particularly interesting because it was done in an internal medicine continuity clinic with the main participation of the internal medicine residents under the supervision of an endocrinologist. It was not done as the majority of the other studies used CGM in specialized endocrinology clinics.

## Introduction

In the United States, there are around 90 million individuals affected by impaired glucose tolerance and/or impaired fasting blood glucose(pre-diabetes), with an additional 34 million diagnosed with diabetes mellitus (DM) [1]. Among those diagnosed, the majority (90-95%) have type 2 diabetes mellitus (T2DM), while 5-10% have type 1 diabetes mellitus (T1DM) or other rare forms of the disease. Furthermore, diabetes ranked as the seventh leading cause of death in the United States in 2019 [1]. Diabetes complications encompass microvascular, macrovascular, and neuropathic issues, such as nephropathy, retinopathy, neuropathy, and cardiovascular events like myocardial infarction and stroke [2].

Successful management of diabetes requires a comprehensive approach involving patient education, active engagement, adherence to dietary restrictions (including carbohydrate and overall caloric control), regular exercise (at least 150 minutes per week), and independent monitoring of glucose levels [3]. Achieving and maintaining proper blood glucose control is crucial in managing diabetes and preventing both immediate and long-term complications associated with hypoglycemia and vascular diseases. The monitoring tools needed to achieve reasonable glycemic control continue to evolve, including more convenient self-monitoring blood glucose (SMBG) meters, continuous glucose monitoring (CGM), and a better understanding of the strengths and limitations of glucose measurement [4-6]. SMBG has been a vital practice for optimizing diabetes therapy in individuals with diabetes, particularly those dependent on insulin therapy, such as individuals with T1DM and T2DM [7-8]. SMBG's limitations in offering a complete profile of blood glucose fluctuation make it inconvenient, while CGM provides individuals with a more comprehensive and detailed overview of their blood glucose levels [9].

This retrospective study seeks to investigate the effectiveness of CGM devices in managing uncontrolled diabetes by assessing changes in various glycemic control parameters, including hemoglobin A1c (HbA1c) levels, average blood glucose levels, hypoglycemic events, time spent within the target blood sugar range, and glucose variability.

## Materials and methods

Study design and setting

This retrospective study was conducted at the internal medicine resident clinic of MountainView Hospital in Las Vegas, Nevada. The study aimed to investigate the effectiveness of CGM devices in managing uncontrolled DM among a cohort of 25 (n = 25) patients, aged between 42 and 75 years. The study team consisted of internal medicine residents, transitional year residents, and a board-certified endocrinologist.

Patient recruitment and consent

Patients with uncontrolled diabetes who predominantly relied on SMBG were informed about CGM devices during their visits to our outpatient clinic. Prior to retrospective data analysis and publication, written consent was obtained from all patients participating in the study. Patients were included based on specific criteria outlined in Table 1.

**Table 1:** Study inclusion and exclusion criteria T1DM: type 1 diabetes mellitus, T2DM: type 2 diabetes mellitus, HbA1c: hemoglobin A1c, SMBG: self-monitoring of blood glucose, CGM: continuous glucose monitoring

Inclusion Criteria	Exclusion Criteria
Patients ages 18-80 years old	Patients who were non-compliant with dietary and exercise recommendations
Patients with the diagnosis of T1DM or T2DM	Patients are unable to understand the instructions for the titration of insulin
Patients with HbA1c >7%	Patient’s wearing their CGM <70% of the time
Patients with uncontrolled blood glucose levels while using SMBG for equal or greater than four times daily	Patients with impaired decision-making capacity
Patients receive their primary care only at the internal medicine resident clinic at MountainView Hospital	Patients missing >2 scheduled visits
Patients can use a CGM device	Patients who are pregnant or incarcerated
Patients on 3-4 subcutaneous injections of insulin +/- oral medications	Patients unresponsive to calls from the clinic
The patient can adjust their insulin based on the CGM data	Patients whose insurance did not cover the CGM

Those patients who did not meet the inclusion criteria were excluded from the trial. Patients expressing interest in CGM devices received detailed explanations regarding their usage, potential risks, and benefits. Additionally, educational pamphlets on CGM device operation, insulin adjustment at home, and counseling on diet and exercise were provided. Patients underwent a teach-back process to ensure their understanding and readiness in operating the CGM device and adjusting their insulin regimen. The Dexcom G6 CGM device (Dexcom, San Diego, California, USA) was used in this study due to its accessibility, reliable technical support, and lack of calibration requirements.

Retrospective follow-up and data collection

In this retrospective study, patients who utilized CGM devices provided by our clinic attended monthly follow-up visits at our internal medicine residency clinic. The patients' monthly clinic notes were thoroughly examined and analyzed retrospectively by our dedicated study team. The primary focus of the analysis was to assess the patients' insulin regimens, which were adjusted based on real-time blood glucose readings obtained from the CGM devices via the Dexcom clarity database. A board-certified endocrinologist played a critical role in providing guidance and oversight, fostering a collaborative approach to implementing timely and personalized modifications with the ultimate goal of optimizing glycemic control for each patient.

Data sources

To collect data, the real-time Dexcom clarity database and our clinic's electronic health records were utilized. Patients with iPhones or Android phones compatible with the CGM device were provided with a share code by the study team, enabling continuous monitoring of their blood glucose levels. For patients without compatible phones using data receiver devices, their CGM device data was retrieved at our clinic. The comprehensive real-time data obtained from the CGM device is presented in Table 2.

**Table 2:** Data evaluated in our study HbA1c: hemoglobin A1c, GMI: glucose management indicator, CGM: continuous glucose monitoring, TIR: time in range

Data Evaluated
Mean HbA1c/GMI before and after implementation of CGM
Average blood glucose
Percentage of TIR before/after implementation of CGM
Mild hypoglycemia is defined as serum blood glucose in the range of 55-70 mg/dl
Severe hypoglycemia is defined as average blood glucose <54 mg/dl
Patients’ medications
Quality of life questionnaire

## Results

The transition from SMBG to CGM devices resulted in significant improvements in glycemic control. Prior to the implementation of CGM, the mean HbA1c level was 11.21%, which significantly improved to 7.04% after CGM introduction, as shown in Table 3.

**Table 3:** Hba1c/GMI before and after the implementation of CGM Hba1c: hemoglobin A1c, GMI: glucose management indicator, CGM: continuous glucose monitoring

	Before CGM device insertion	After CGM device insertion
Patients’ average HbA1c/GMI	11.21%	7.04%

Similar positive effects were observed in average blood glucose levels, reduction in hypoglycemic events, and increased time spent within the target range, as shown in Table 4.

**Table 4:** TIR and coefficient of variation results in SMBG vs. CGM TIR: time in range, SMBG: self-monitoring of blood glucose, CGM: continuous glucose monitoring

	SMBG	CGM
TIR	18%	74%
Coefficient of variation	39%	29%

This improvement was accompanied by a reduction in average blood glucose levels from 286 mg/dl to 158 mg/dl. The occurrence of hypoglycemic events also decreased, with mild hypoglycemia episodes decreasing from 4.75% to 0.78% and pronounced hypoglycemia episodes decreasing from 3.01% to 0.2%. Furthermore, the time in range (TIR) improved from 18% with SMBG to 74% with the CGM device. The CoV, a measure of glucose variability, decreased from 39% to 29% after CGM implementation, meeting the goal of below 36%. Notably, a subgroup of patients with T2DM (16%) was able to discontinue their rapid-acting insulin before meals and effectively manage their condition solely with oral anti-diabetic medications and/or weekly injectable GLP-1 receptor agonists after transitioning from SMBG to CGM.

These results demonstrate the positive impact of CGM devices on glycemic control, highlighting their potential to improve patient outcomes in diabetes management. There were no other variables that could affect our good results besides the device used.

## Discussion

The management of diabetes has been continuously evolving over time. In the past, various methods were employed for diagnosing DM and monitoring blood glucose levels [10-12]. In the 1970s, Anton Clemens introduced the first blood glucose meter, revolutionizing point-of-care testing with significant advancements in the field [13]. Studies like the UK Prospective Diabetes Study and the Diabetes Control and Complications Trial emphasized the importance of blood glucose monitoring in intensive diabetes treatment [14]. SMBG has become a vital part of diabetes care since its recommendation by the American Diabetes Association (ADA) in 1987 [15]. SMBG empowers patients to manage their condition effectively, improving outcomes and achieving A1c goals [16]. However, challenges such as the burden of adherence to SMBG four times per day, cost, and regulatory issues persist from both patients' and manufacturers' perspectives [10].

Patients often encounter challenges in effectively adhering to SMBG, including the responsibility of self-care, the discomfort of regular fingerprick tests, factors like ethnicity, anxiety, perceptions of diabetes, vulnerability to complications, and the quality of support from healthcare providers and family [10,17-18]. In 1999, a groundbreaking advancement in diabetes care unfolded with the approval of the first-ever CGM system, marking the onset of a new era in assisting individuals newly diagnosed with diabetes [19]. The CGM devices have transformed diabetes care by allowing patients to continuously monitor their blood sugars through subcutaneously inserted devices, providing comprehensive and frequent data that enables medical providers to make personalized adjustments to drug therapy based on individual patients' unique glucose fluctuations and lifestyles [20].

The CGM devices offer a significant advantage over SMBG by providing a wealth of time-series glucose data, allowing up to 288 glucose values per day, revealing temporal trends and patterns in glucose control, and enhancing the detection of asymptomatic hypoglycemia [21]. Furthermore, CGM devices allow patients to set personalized alarms for glucose levels that surpass or fall below specific thresholds, thereby promoting timely intervention and providing significant advantages for individuals, particularly youths and parents, in the management of diabetes [22-23]. CGM benefits are well-established in T1DM, but limited research exists for T2DM patients, with short study durations [24]. CGM demonstrated improved outcomes in T1DM with lower HbA1c and reduced severe hypoglycemia, while in T2DM, CGM significantly lowered HbA1c compared to SMBG based on meta-analysis results [25-26].

The results of our study demonstrated that the adoption of CGM device leads to significant improvements in glycemic control, as evidenced by a decrease in mean HbA1c levels from 11.21% to 7.04% and a reduction in average blood glucose levels from 286 mg/dl to 158 mg/dl. Additionally, the occurrence of hypoglycemic events decreased, with mild hypoglycemia decreasing from 4.75% to 0.78% and severe hypoglycemia decreasing from 3.01% to 0.2%. These findings align with the ADA's goals, demonstrating the effectiveness of CGM in achieving glycemic targets (R). CGM also improved TIR, with patients spending 74% of the time within the target blood sugar range of 70-180 mg/dl, exceeding the ADA's recommended goal of 70%. Furthermore, CGM usage increased the time spent within the target blood sugar range, meeting or exceeding recommended goals, and reduced glucose variability.

There were three patients with T2DM who were able to gradually eliminate their reliance on insulin therapy and successfully transitioned to alternative treatments such as oral anti-diabetic medications or GLP1 agonists once-a-week subcutaneous injections. This significant outcome highlights the effectiveness of CGM in glycemic control and suggests the potential for reducing reliance on insulin therapy while achieving improved patient outcomes in the management of T2DM.

It is important to note that our study had a limited sample size and short duration of follow-up and lacked a control group for comparison. There may be potential selection bias, a lack of long-term follow-up, and limited diversity among participants. Self-reported data and potential confounding factors were not fully addressed. The study did not include a cost-effectiveness analysis and was conducted in a single healthcare center. It is important to acknowledge these limitations and consider them when interpreting the results of our study. Future research should aim to address these limitations to provide a more comprehensive understanding of the benefits and challenges associated with CGM device usage in diabetes management. Further large-scale studies are essential to validate these findings.

## Conclusions

In conclusion, our study demonstrates that CGM devices lead to significant improvements in glycemic control in comparison to SMBG, including reductions in HbA1c levels, average blood glucose levels, and hypoglycemic events. CGM usage also increases time spent within the target blood sugar range and reduces glucose variability. These findings support the efficacy of CGM in optimizing glycemic control and its feasibility in real-world clinical practice. Further research is needed to validate these results and explore CGM's broader impact.

Moreover, it is important to highlight that the patients enrolled in this study were under the care of residents at an internal medicine continuity clinic. This significant observation suggests that the successful implementation of CGM is feasible within resident clinics, emphasizing its potential benefits in real-world clinical practice. To further establish and expand upon this approach, it is imperative to secure funding for similar pilot programs with governmental support. This research would serve to assess the broader impact and applicability of CGM devices in diabetes management. Large-scale randomized controlled longitudinal studies are needed to validate our research results and confirm our concept that improvement of DM control can be achieved using a CGM device in an internal medicine continuity clinic operated by internal medicine residents with the supervision of an endocrinologist when needed and not only in specialized endocrine clinics.
